# Interleukin-33 regulates hematopoietic stem cell regeneration after radiation injury

**DOI:** 10.1186/s13287-019-1221-1

**Published:** 2019-04-18

**Authors:** Ping Huang, Xiangyong Li, Ying Meng, Baohong Yuan, Tao Liu, Mengya Jiao, Xiaodi Wang, Yunjun Liu, Hui Yin

**Affiliations:** 1 Department of Microbiology and Immunology, Guangdong Pharmaceutical University, Guangzhou, 510006 China; 2 Institute of Biochemistry and Molecular Biology, Guangdong Medical University, Zhanjiang, 524023 China; 3 Guangdong Provincial Key Laboratory of Pharmaceutical Bioactive Substances, Guangdong Pharmaceutical University, Guangzhou, 510006 China; 4 School of Pharmacy, Guangdong Pharmaceutical University, Guangzhou, 510006 China

**Keywords:** IL-33, Ionizing radiation, Hematopoietic stem cells, Cell apoptosis, PUMA

## Abstract

**Background:**

IL-33 is a pleiotropic cytokine of the IL-1 family, which has been reported to implicate in both innate and adaptive immune responses. Recent studies suggest IL-33 is crucial for regulation of myelopoiesis and myeloid cell activity. Here, we explore the potential effect of IL-33 against hematopoietic injury after total body irradiation (TBI).

**Methods:**

C57BL/6 mice were irradiated with a sublethal dose of radiation (600 cGy) and treated with IL-33 at a dose of 3 μg/dose i.p. once a day for seven consecutive days. H&E staining was used to determine the bone marrow cellularity. A flow cytometer was used to quantify the hematopoietic stem cell (HSC) population, cell proliferation, and apoptosis. The colony-forming assay was used to evaluate the clonogenic function of HSCs. RT-qPCR was used to determine the expression of apoptosis-associated genes.

**Results:**

Bone marrow HSCs from wild-type mice expressed functional IL-33 receptor (ST2), and treatment with IL-33 promoted the recovery of the HSC pool in vivo and improved the survival of mice after TBI. Conversely, mice with ST2 deficiency showed decreased HSC regeneration and mouse survival after TBI. Of note, IL-33 reduced radiation-induced apoptosis of HSCs and mediated this effect through repression of the p53-PUMA pathway.

**Conclusions:**

IL-33 regulates HSC regeneration after myelosuppressive injury through protecting HSCs from apoptosis and enhancing proliferation of the surviving HSCs.

## Background

When an acute radiation catastrophe occurred owing to a nuclear detonation, terrorist radiological attack, or a nuclear power plant accident, the first responders and civilians are at high risk of exposure to lethal doses of ionizing radiation (IR) [1, 2]. The acute radiation syndrome (ARS) is characterized by a series of complex physiological and morphological developments manifesting eventually in multi-organ failure (MOF) resulting in the death of the casualties [3]. The hematopoietic system is one of the most sensitive tissues to IR, and transient myelosuppression involves loss of BM cellularity and damage to hematopoietic progenitor cells at moderate doses [4, 5]. Moreover, IR doses beyond 3.5 Gy can lead to BM failure owing to a severe injury to hematopoietic stem cells (HSCs) that can transform into long-term BM damage on complete ablation of HSC reserves and functions [6]. Hence, recovery and survival following exposure to myeloablative doses of total body irradiation (TBI) is primarily dependent on the maintenance of HSC homeostasis, self-renewal capability and their ability to stimulate requisite levels of immune compartments [7, 8].

Interleukin-33 is a newly described member of the IL-1 family of cytokines that binds to the IL-33 receptor, formerly known as the orphan receptor ST2 [9, 10]. Like the members of IL-1 family IL-1β and IL-18, IL-33 is produced as a 31-kDa precursor that is cleaved by caspase 1 to form a mature protein [11]. IL-33 has been reported to be constitutively expressed in the nuclei of endothelial and epithelial cells, and is released into the extracellular space, as an alarmin, after tissue damage to alert the immune system [12]. Incipient studies have reported that IL-33 is a strong inducer of pro-inflammatory cytokines and chemokines by mast cells [13], resulting in the development or exacerbation of asthma or atopic allergy and anaphylaxis [14]. In addition to acting as an alarmin or inducer of pro-inflammatory reaction, IL-33 also plays a crucial role during allograft survival via the initiation of Th2-type immune response or promotes neutrophil recruitment and phagocytosis against severe sepsis [15–17]. Recent emerging evidence showed that the IL-33 receptor ST2 is expressed on the surface of HSCs and various subsets of HPCs in mice and human HPCs produce IL-5, IL-13, and chemokines in response to IL-33 either alone or in combination with other inflammatory stimuli [18, 19]. However, the role of IL-33 on modulation of HSC homeostasis remains poorly understood.

In the present study, we tested the radioprotective function of IL-33 against hematopoietic damage caused by ionizing radiation. Our results showed that the expression of the IL-33 receptor ST2 on the surface of HSCs was rapidly increased in mice when exposed to ionizing radiation. Exogenous delivery of IL-33 apparently improved the survival of mice after sublethal doses of ionizing radiation, whereas genetic deficiency of ST2 antagonized hematopoietic reconstitution in vivo. More importantly, the radioprotection of IL-33 in the hematopoietic failure was involved in the inhibition of HSC apoptosis and promotion of HSC regeneration.

## Methods

### Mice and reagent

Seven- to 10-week-old male ST2-deficient mice on a C57BL/6 background and their wild-type littermates were obtained from Cyagen Biosciences Inc., Guangzhou, China. All animals were housed in specific pathogen-free conditions, and experiments were approved by the Animal Care and Use Committee of Guangdong Pharmaceutical University. Recombinant mouse IL-33 protein was prepared in our laboratory as described previously [20].

### X-ray irradiation and IL-33 treatment

Mice were sublethally irradiated with 600 cGy (at a dose rate of 100 cGy/min) TBI using an RS2000 X-ray irradiator (RAD Source Technologies, Coral Springs, FL, USA). For evaluation of the radiation-mitigating efficacy of IL-33, irradiated mice were injected i.p. with IL-33 (3.0 μg/mouse) or phosphate-buffered saline (PBS) daily for 7 days. Results were plotted on a Kaplan-Meier survival curve.

### Transplantation assay

WT mice were lethally irradiated with 900 cGy TBI and transplanted with 5 × 10^5^ BM cells from donor mice. The survival rates of the mice were monitored for 3 months. Ten weeks after transplantation, bone marrow cells of the recipients were stained with anti-c-kit, anti-Sca-1, and anti-lineage cocktail antibodies (BioLegend, San Diego, CA, USA) to measure c-Kit^+^Sca-1^+^Lin^−^ (KSL) progenitor cells by a FACSCalibur cytometer (BD Biosciences, San Jose, CA, USA).

### In vitro cultures with primary HSC cells

Mouse bone marrow Lin^−^c-Kit^+^ cells were obtained by a combination of negative selection for Lin^−^ cells and positive selection for c-Kit^+^ cells using MicroBeads (Miltenyi Biotec Inc., Auburn, CA, USA) following the protocols provided by the manufacturer. They were seeded into wells of 24-well plates at 3 × 10^5^ cells per well and incubated with StemSpan™ SFEM (StemCell Technologies, Vancouver, Canada) supplemented with l-glutamine, penicillin-streptomycin (Invitrogen), and three cytokines: 20 ng/ml thrombopoietin, 125 ng/ml stem cell factor, and 50 ng/ml Flt-3 ligand (TSF; R&D Systems, Minneapolis, MN, USA). Some wells were supplemented with TSF alone or TSF with 50 ng/ml IL-33 following 200 cGy for 72 h and then collected for total cell counts and CFCs analysis. Some wells were cultured with TSF alone, TSF with 50 ng/ml IL-33, or TSF, IL-33, and 1 μM Ly294002 (Selleck), following 200 cGy for 30 min or 72 h and then collected for phosphorylation AKT-S473 analysis or CFCs assay, respectively. Other wells were cultured with TSF alone, TSF with 50 ng/ml IL-33, or TSF, IL-33, and P53 inhibitor Pifithrin-α (10 μM; Selleck), following 200 cGy for 72 h, and then collected for cell apoptosis analysis.

### HSC colony formation assays

For in vitro colony formation assays, either whole BM or cultured Lin^−^c-Kit^+^ cells were plated onto MethoCult GF 3534 medium (StemCell Technologies, Vancouver, Canada) and colonies were scored on day 12. Experiments to assess colony-forming unit-spleen day 12 (CFU-S12) was performed following the protocol described previously [21]. 2 × 10^5^ BM cells were collected from donor mice and injected via tail vein into recipient C57BL/6 mice that had been given 900 cGy TBI. Spleens from recipient mice were harvested after day 12 irradiation and preserved in Bouin’s solution for counting the colonies.

### Lentivirus transduction

Purified Lin^−^c-Kit^+^ cells were infected with either PUMAα shRNA lentiviral particles (PUMA shRNA LV) or control shRNA LV (Santa Cruz Biotechnology) in StemSpan™ SFEM with 5 μg/ml polybrene at multiplicities of infection (MOI) of 15. After 12 h of culturing, the medium was replaced by fresh SFEM in order to remove debris and inactive lentiviruses. Some wells were then supplemented with IL-33 (50 ng/mL) following 200 cGy for another 72 h and then collected for cell apoptosis and CFC analysis.

### Flow cytometry

BM cells were flushed from the femurs of mice with PBS containing 2% fetal bovine serum (FBS), and the red blood cells were lysed with lysing buffer (eBioscience, San Diego, CA, USA). To examine the percentages of ST2^+^KSL cells, BM cells were suspended in PBS and incubated with PE-labeled anti-ST2, FITC-conjugated antibodies specific for lineage markers (CD3e, B220, TER119, CD11b, and Gr-1), PerCP-Cy5.5-conjugated anti-c-Kit, and APC-conjugated anti-Sca-1 antibodies (BioLegend) for 30 min. Cell apoptosis was measured using an annexin V apoptosis detection kit according to the instructions of the manufacturer (eBioscience). For analysis of phosphorylation AKT-S473, cells were fixed and permeabilized with the BD Cytofix/Cytoperm kit (BD Biosciences) and then stained with mouse anti-phospho-AKT-S473 PE or isotype control (eBioscience). For analysis of PUMA or p53 protein levels, cells were fixed in 4% paraformaldehyde and permeabilized in 0.25% saponin. Cells were stained with a primary anti-PUMA or anti-p53 antibody (MultiSciences Biotech Co., Ltd., Hangzhou, China) followed by a secondary donkey anti-rabbit PE antibody (BioLegend). Flow cytometric analysis was performed with FACSCalibur cytometer and CellQuest v3.3 software.

### Cell proliferation

For analysis of HSC cell proliferation in vitro, Lin^−^c-Kit^+^ cells were cultured with TSF alone or TSF with 50 ng/ml IL-33 following 200 cGy for 72 h, fixed, and permeabilized using the BD Cytofix/Cytoperm kit and stained intracellularly with PE anti-Ki-67 (eBioscience). To monitor the proliferating hematopoietic cells in vivo, the mice were intravenously injected with BrdU (100 mg/kg body weight) 24 h before sacrifice. BM cells were labeled with anti-lineage FITC, anti-Sca-1 APC, anti-c-Kit PerCP-Cy5.5, and anti-BrdU PE (eBioscience). Incorporation of BrdU was analyzed by flow cytometry according to the manufacturer’s staining protocol.

### Cell-cycle analysis

The cell was fixed with 75% ethanol at 4 °C for 16 h and then treated with 100 μg/mL ribonuclease A and 50 μg/mL propidium iodide (PI; Sigma, St Louis, MO, USA) at room temperature for 30 min. DNA fluorescence of the stained cells was measured with a FACSCalibur cytometer. The percentages of cells within the G1, S, and G2/M phases of the cell cycle were calculated by use of ModFit software (Verity, Topsham, ME).

### Histology

Mice were sacrificed and femurs isolated on day 7 post treatment. Femurs were fixed in 10% neutral-buffered formalin followed by decalcification with 10% EDTA for 4–5 days and then embedded with paraffin. Five-micrometer-thick sections were made and stained with hematoxylin and eosin (H&E). The slides were observed by optical microscopy (Leica) to capture bright-field images.

### Enzyme-linked immunosorbent assay (ELISA)

The whole bone marrow was collected from mice and at 7 days following 600 cGy TBI. After centrifugation, BM supernatants were collected and analyzed for cytokine concentrations using ELISA kit (eBioscience), according to the manufacturer’s guidelines.

### Real-time quantitative PCR (RT-qPCR)

Total RNA was extracted from samples using TRIzol reagent (Invitrogen), and reverse transcription was made using the first strand cDNA synthesis kit (Invitrogen). RT-qPCR was performed to detect mRNA expression using SYBR Green qPCR kit (Invitrogen), and primers used in the PCR amplification were p53 forward 5′-TCACAGTCGGATATCAGCCT-3′, reverse 5′-ACACTCGGAGGGCTTCACTT-3′; PUMA forward 5′- CCTCCTTTCTCCGGAGTGTTCA-3′, reverse 5′- ATACAGCGGAGGGCATCAGG-3′; and GAPDH forward 5′-TTC ACC ACC ATG GAG AAG GC-3′, reverse 5′-GGC ATG GAC TGT GGT CAT GA-3′. All RT-qPCR reactions were performed with an ABI PRISM® 7000 Sequence Detector Systems (Applied Biosystems, Foster City, CA, USA), and expression values were normalized to the housekeeping gene GAPDH using the comparative threshold cycle (CT) method.

### Statistical analyses

All the data are presented as the mean ± standard error of the mean (SEM). Statistical differences between groups were evaluated by the Student’s *t* test or one way ANOVA. Survival data were analyzed by the log-rank test. *P* values less than 0.05 were considered significant.

## Results

### IL-33 administration improves the survival of irradiated mice

**Fig. 1:**
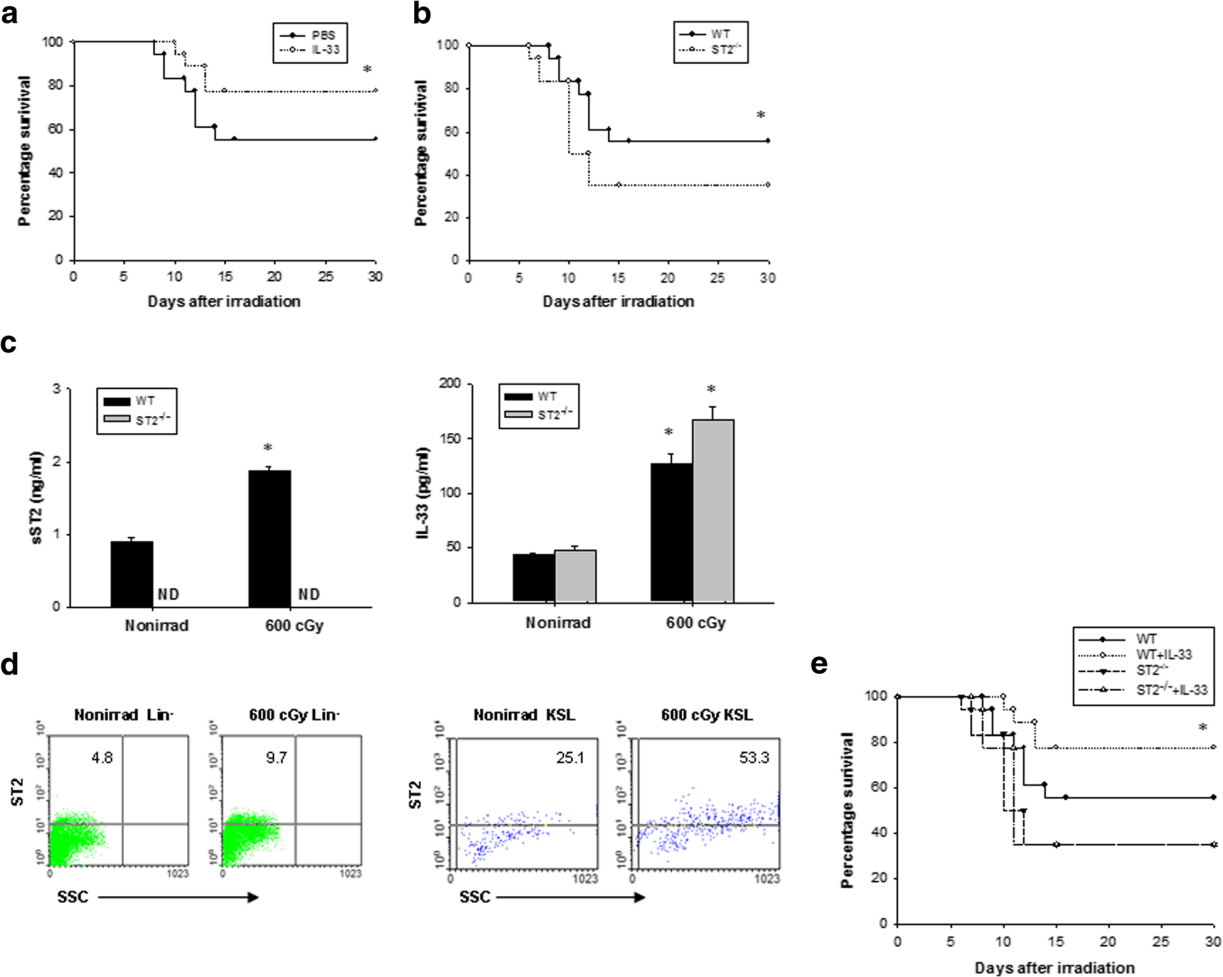
IL-33 administration improves survival of mice after TBI. **a** Survival curves of C57BL/6 mice that were irradiated with 600-cGy TBI followed by daily IL-33 or PBS treatments for 7 days. Data are pooled from three experiments, *n* = 6 mice per group per experiment. *P < 0.05 compared to untreated controls. **b** Survival rate of untreated *ST2*^*−/−*^ and WT mice was irradiated with 600-cGy. Data are pooled from three experiments, *n* = 6 mice per group per experiment. *P < 0.05 compared to *ST2*^*−/−*^ controls. **c** sST2 and IL-33 concentration in the bone marrow serum of WT and *ST2*^*−/−*^ mice before irradiation (Nonirrad) and at 7 days after 600-cGy irradiation. Data are mean ± SEM (*n* = 6 in each group). *P < 0.05 compared to nonirradiated controls. **d** Representative ST2 surface expression on bone marrow Lin^−^ cells and KSL cells from C57BL/6 mice before irradiation and at 6 h after 600-cGy irradiation. The numbers shown indicate the percentage of ST2 surface expression on the indicated cell population. **e** Survival rate of *ST2*^*−/−*^ and WT mice was irradiated with 600-cGy and treated with IL-33 as in **a**

We first explore whether systemic administration of IL-33 could improve the survival of sublethal irradiation. C57BL/C mice were whole-body irradiated with 600 cGy and treated with IL-33 i.p. at a dose of 3 μg/dose/day once a day for 7 consecutive days starting within 1 h after radiation exposure. As shown in Fig. 1a, 77% of IL-33-treated mice survived more than 30 days post radiation in comparison with 55% of irradiated controls.

To demonstrate an endogenous role and specificity of IL-33, we performed an equal dose of TBI in ST2 knockout (*ST2*^*−/−*^) mice. *ST2*^*−/−*^ mice showed a higher mortality rate than WT mice when explored to 600 cGy TBI (Fig. 1b). The WT mice after TBI produced substantial amounts of sST2 and IL-33 in the bone marrow serum, whereas the *ST2*^*−/−*^ TBI mice produced higher concentrations of IL-33 than the WT TBI mice (Fig. 1c). Less than 5% of bone marrow Lin^−^ cells expressed ST2, but 25% of bone marrow KSL cells expressed ST2. ST2 surface expression increased twofold in bone marrow KSL cells at 6 h after 600 cGy TBI (Fig. 1d). Moreover, IL-33 reduced the mortality in WT mice but not *ST2*^*−/−*^ mice (Fig. 1e). Together, these data suggest that IL-33 possesses a radioprotective effect via IL-33–ST2 signaling.

### IL-33 treatment promotes HSC regeneration in vivo

**Fig. 2:**
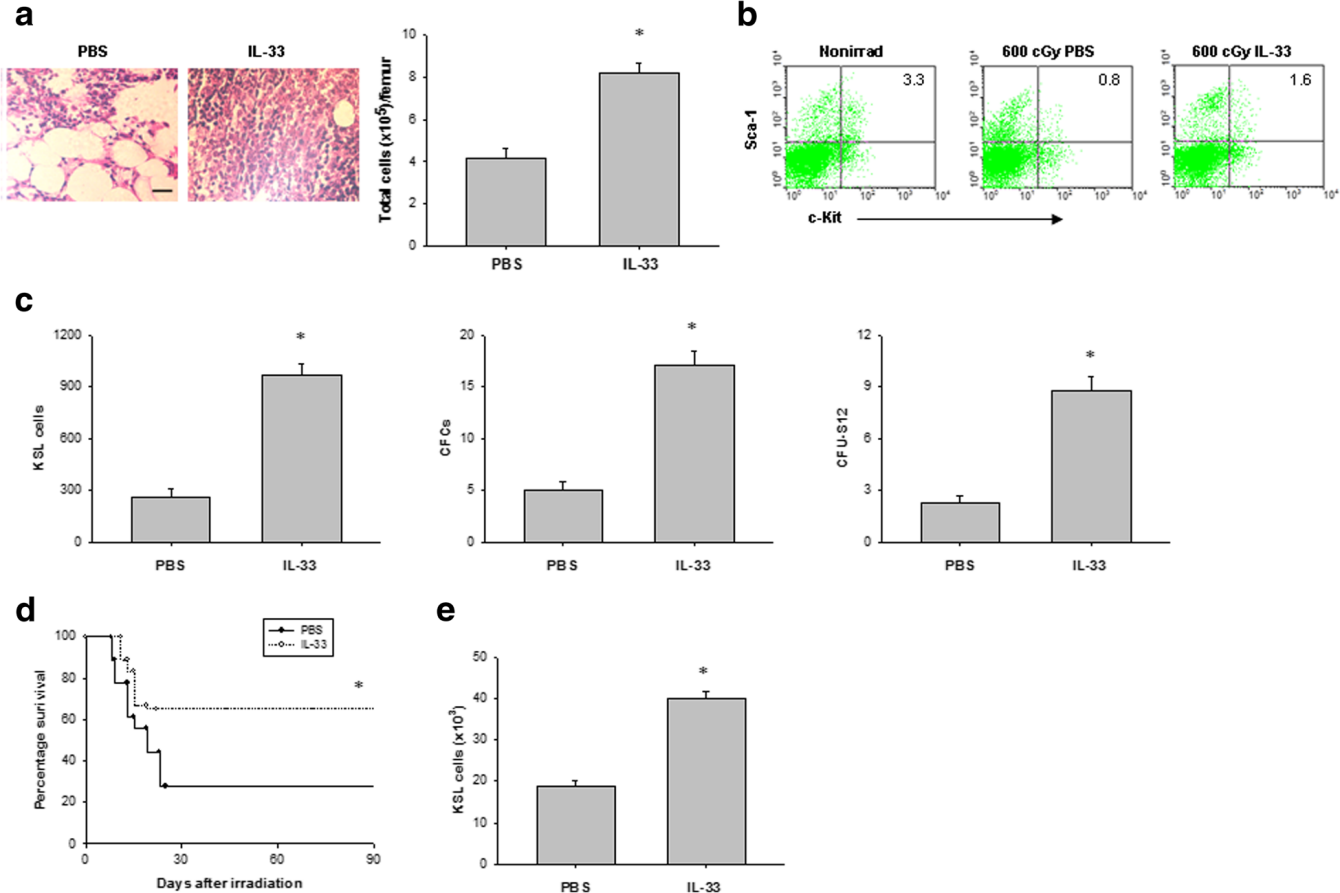
IL-33 signaling mediates HSC regeneration in vivo. **a** Left, representative H&E-stained femurs from irradiated mice treated with either PBS or IL-33 for 7 days. Scale bar, 100 μm. Right, bone marrow cell counts. Data are mean ± SEM (*n* = 6 in each group). *P < 0.05 compared to untreated controls. **b** Representative FACS analysis of bone marrow c-Kit^+^Sca-1^+^ cells within the Lin^−^ gate (KSL) from nonirradiated (Nonirrad) mice and at day 7 from irradiated mice treated with either PBS or IL-33. The numbers shown indicate the percentage of c-Kit^+^Sca-1^+^ cells within the Lin^−^ population. **c** Bone marrow KSL cells, CFCs, and CFU-S12 at day 7 in irradiated mice treated with either PBS or IL-33. Data are mean ± SEM (*n* = 6 in each group). *P < 0.05 compared to untreated controls. **d** Survival rates of lethally irradiated mice that were adoptively transferred with 5 × 10^5^ BM cells from irradiated mice treated with or without IL-33. Data are pooled from three experiments, *n* = 6 mice per group per experiment. *P < 0.05 compared to untreated controls. **e** KSL cells in the BM of recipient mice at 10 weeks after transplantation of 5 × 10^5^ bone marrow cells from irradiated and PBS-treated or irradiated and IL-33-treated donor mice. Data are mean ± SEM (*n* = 6 in each group). *P < 0.05 compared to untreated controls

To explore whether IL-33 treatment can promote HSC regeneration in vivo, we measured hematopoietic reconstitution in C57BL/6 mice after 600 cGy TBI. Histological analyses of the bone marrow suggested that IL-33 increased the cellularity of the bone marrow at day 7 post irradiation, as well as enhanced numbers of bone marrow cells compared to PBS-treated controls (Fig. 2a). To further understand the effect of IL-33 in hematopoiesis, we also measured hematopoietic progenitor cells in the bone marrow. As shown in Fig. 2b, c, IL-33 significantly increased the numbers of bone marrow KSL cells, colony-forming cells (CFCs), and CFU-S12 compared with irradiated controls.

To determine whether IL-33 administration can accelerate the hematopoietic reconstitution in irradiated mice, we transplanted lethally irradiated recipient mice with bone marrow cells from donor mice irradiated with 600 cGy and treated with either IL-33 or PBS. Sixty-five percent of mice transplanted with 5 × 10^5^ BM cells from irradiated and IL-33-treated donors survived more than 90 days, compared with less 30% of transplanted control mice (Fig. 2d). At 10 weeks after transplant, mice transplanted with 5 × 10^5^ BM cells from IL-33-treated donors showed high KSL cells in the bone marrow compared to mice transplanted with the bone marrow from irradiated and PBS-treated donors (Fig. 2e). All together, these findings imply that IL-33 treatment significantly increased the recovery of bone marrow HSCs in mice after TBI.

### IL-33 regulates HSC proliferation after irradiation

**Fig. 3:**
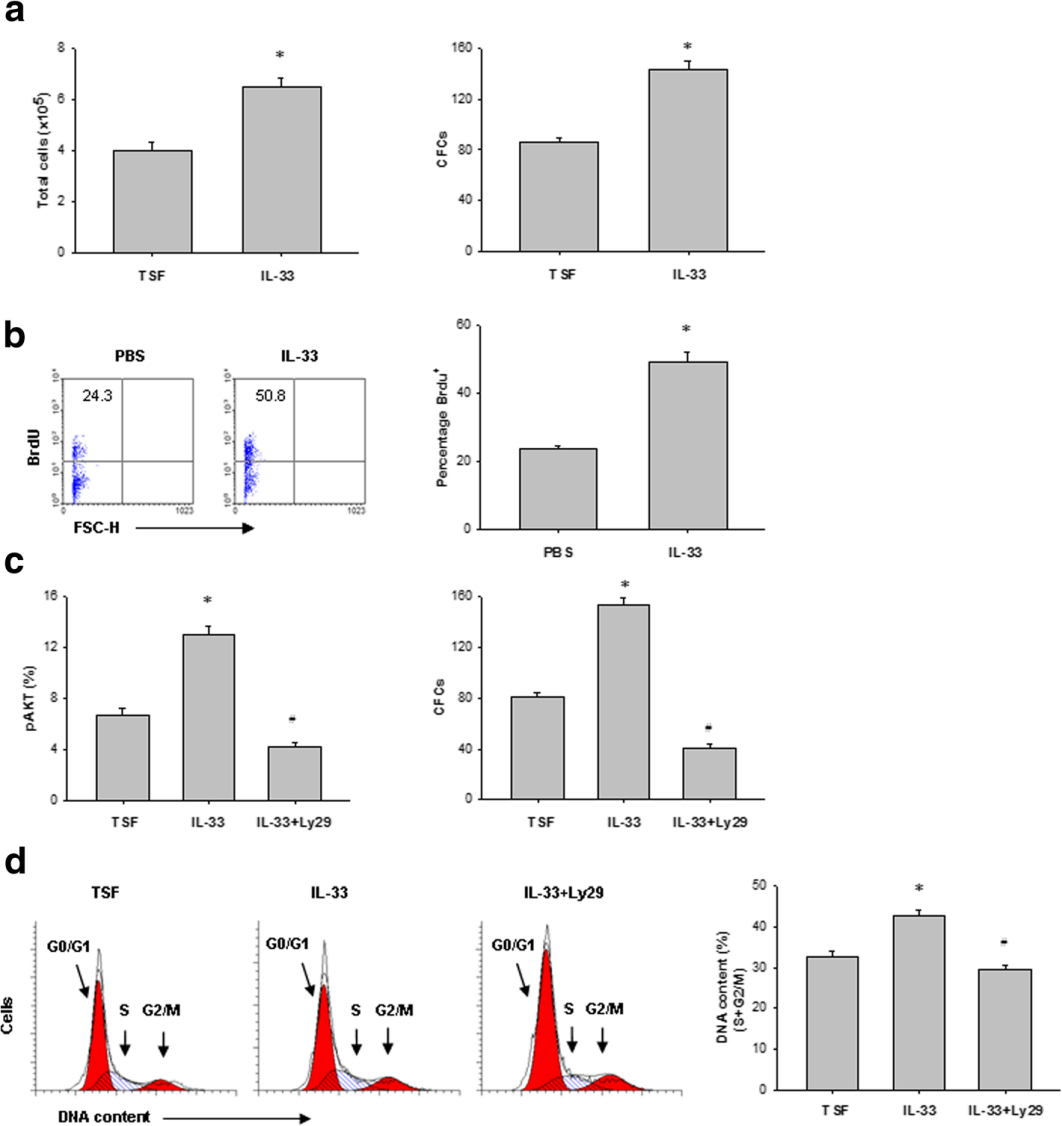
IL-33 regulates HSC proliferation after irradiation. **a** Numbers of total cells and CFC at 72 h in irradiated bone marrow Lin^−^c-Kit^+^ cells cultured with either TSF or TSF plus IL-33. Data are mean ± SEM (*n* = 3 in each group). *P < 0.05 compared to untreated controls. **b** Representative BrdU incorporation in bone marrow KSL cells in vivo at day 7 after 600-cGy TBI and treatment with either PBS or IL-33 (left). Right, numbers indicate the percentage of BrdU^+^ cells within the total bone marrow KSL population. Data are mean ± SEM (*n* = 3 in each group). *P < 0.05 compared to untreated controls. **c** Left, percentage phosphorylated AKT (pAKT) in bone marrow Lin^−^c-Kit^+^ cells after 200-cGy irradiation and the culture conditions are shown. Right, CFCs from bone marrow Lin^−^c-Kit^+^ cells after 200-cGy irradiation and the culture conditions are shown. *P < 0.05 for TSF compared to IL-33, ^#^*P* < 0.01 for IL-33 compared to IL-33 plus Ly294002 (Ly29) (mean ± SEM, *n* = 3 in each group). **d** Representative FACS analysis of the cell-cycle status of bone marrow Lin^−^c-Kit^+^ cells at 72 h after irradiation and the culture conditions shown. *P < 0.05 for TSF compared to IL-33, ^#^*P* < 0.01 for IL-33 compared to IL-33 plus Ly294002 (Ly29) (mean ± SEM, *n* = 3 in each group)

We next sought to determine the effects of IL-33 on HSC proliferation and survival after radiation exposure in vitro. Hematopoietic cells were irradiated with 200 cGy and cultured with TSF alone or TSF plus IL-33. The total cell number and CFC were determined after 3 days in culture. As shown in Fig. 3a, IL-33 significantly increased the irradiated cell number compared to TSF treatment alone. Total number of CFC in irradiated bone marrow cells were also significantly increased in the presence of IL-33. In addition, IL-33 treatment of mice for 7 days after 600-cGy TBI also significantly increased BrdU incorporation in bone marrow KSL cells compared to PBS-treated irradiated mice (Fig. 3b).

Because IL-33-mediated cell proliferation is known to involve phosphatidylinositol-4,5-bisphosphate 3-kinase (PI3K)-AKT pathways [22], we next looked for evidence of this signaling mechanism in our experiments. In irradiated bone marrow Lin^−^c-Kit^+^ cells, IL-33 treatment increased AKT phosphorylation corresponded with enhanced CFC recovery (Fig. 3c). Treatment of irradiated bone marrow Lin^−^c-Kit^+^ cells with Ly294002, a PI3K inhibitor, blocked IL-33-mediated AKT phosphorylation and inhibited bone marrow progenitor cell recovery in response to IL-33 (Fig. 3c). Irradiated bone marrow Lin^−^c-Kit^+^ cells treated with IL-33 and Ly294002 also showed significantly decreased cell cycling compared to Lin^−^c-Kit^+^ cells treated with IL-33 alone (Fig. 3d). All together, these data suggest that IL-33 treatment contributes to HSC cycling and progenitor cell recovery, which is related to, at least in part, activation of the PI3K-AKT pathway.

### IL-33 protects HSCs from apoptosis after irradiation

**Fig. 4:**
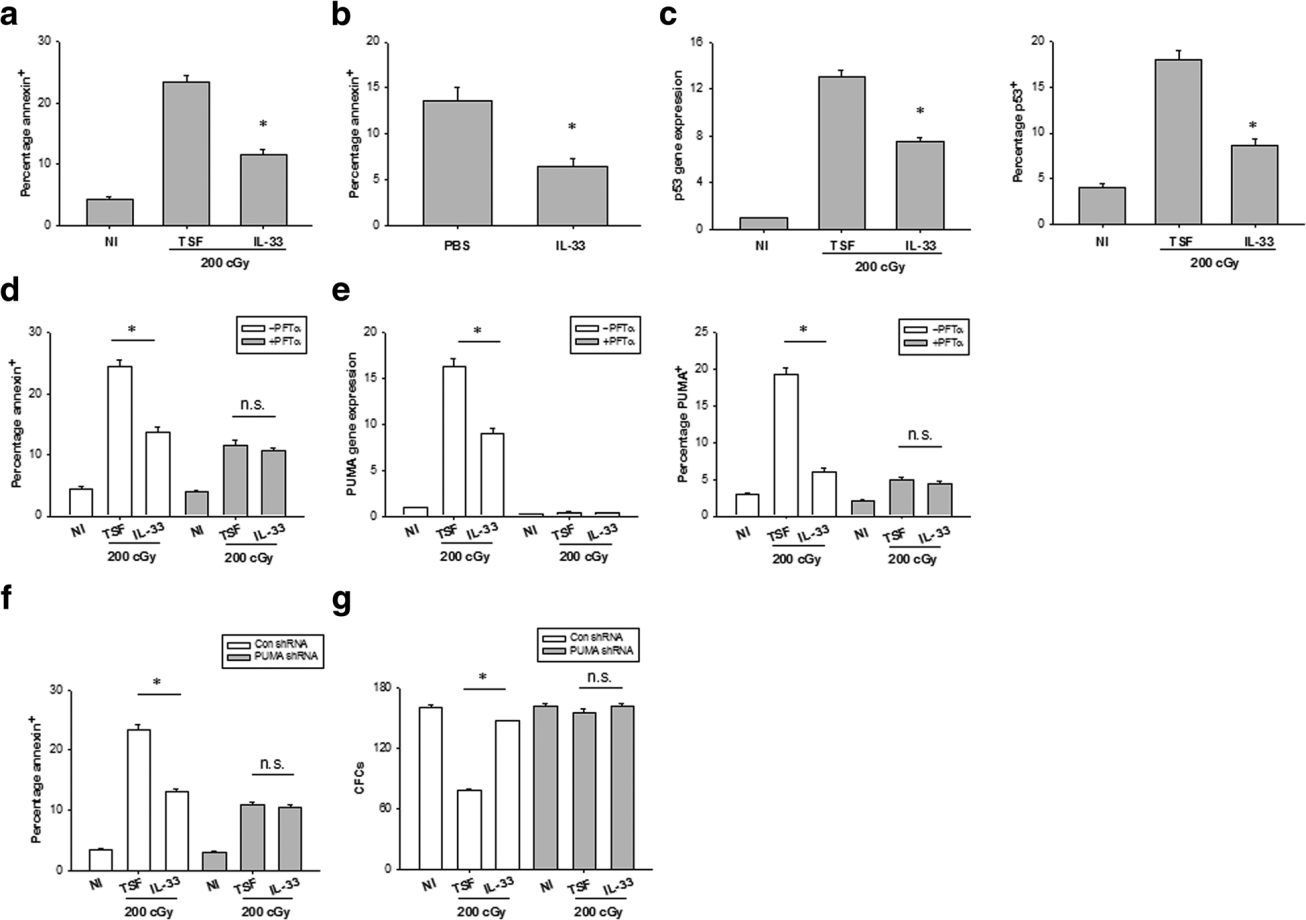
IL-33 protects HSCs from apoptosis after irradiation. **a** The percentage of annexin^+^ bone marrow Lin^−^c-Kit^+^ cells at 72 h of culture with TSF or TSF plus IL-33 and after 200-cGy irradiation and the culture conditions are shown. Data are mean ± SEM (*n* = 3 in each group). *P < 0.05 compared to untreated controls. **b** The percentage of annexin^+^CD45^+^ cells in the bone marrow at day 7 after 600-cGy TBI and treatment with PBS or IL-33. Data are mean ± SEM (*n* = 6 in each group). *P < 0.05 compared to untreated controls. **c** Left, P53 mRNA expression in bone marrow Lin^−^c-Kit^+^ cells at 6 h of culture with TSF or TSF plus IL-33 and after 200-cGy irradiation and the culture conditions are shown. Right, mean percentages of P53 protein expression in Lin^−^c-Kit^+^ cells at 36 h of culture with TSF or TSF plus IL-33 and after 200-cGy irradiation and the culture conditions are shown. Data are mean ± SEM (*n* = 3 in each group). *P < 0.05 compared to untreated controls. **d** The percentage of annexin^+^ Lin^−^c-Kit^+^ cells with or without P53 inhibition at 72 h of culture with TSF or TSF plus IL-33 and after 200-cGy irradiation and the culture conditions are shown. Data are mean ± SEM (*n* = 3 in each group). *P < 0.05 compared to untreated controls. n.s. not significant. **e** Left, PUMA mRNA expression in bone marrow Lin^−^c-Kit^+^ cells at 6 h of culture with TSF or TSF plus IL-33 and after 200-cGy irradiation and the culture conditions are shown. Right, mean percentages of PUMA protein expression in Lin^−^c-Kit^+^ cells at 36 h of culture with TSF or TSF plus IL-33 and after 200-cGy irradiation and the culture conditions are shown. Data are mean ± SEM (*n* = 3 in each group). *P < 0.05 compared to untreated controls. n.s. not significant. **f** The percentage of annexin^+^ Lin^−^c-Kit^+^ cells with or without PUMA knockdown at 72 h of culture with TSF or TSF plus IL-33 and after 200-cGy irradiation and the culture conditions are shown. Data are mean ± SEM (*n* = 3 in each group). *P < 0.05 compared to untreated controls. n.s. not significant. **g** CFCs from bone marrow Lin^−^c-Kit^+^ cells with or without PUMA knockdown in irradiated TSF culture and irradiated TSF plus IL-33 culture groups. Data are mean ± SEM (*n* = 3 in each group). *P < 0.05 compared to untreated controls. n.s. not significant

We further assess whether the effect of IL-33 on hematopoietic recovery is involved in protection against HSC apoptosis after irradiation. At 72 h after 200 cGy irradiation, IL-33-treated cultures contained twofold decreased numbers of annexin-positive Lin^−^c-Kit^+^ cells compared to alone cytokine cultures (Fig. 4a). C57BL/6 mice irradiated with 600 cGy and then treated with IL-33 for 7 days displayed more than twofold decreased numbers of annexin-positive bone marrow hematopoietic cells compared to PBS-treated controls (Fig. 4b).

It was well known that P53-PUMA pathway is implicated in radiation-induced hematopoietic toxicity [23]. Because IL-33 promoted HSC survival after irradiation, we examined whether IL-33 causes such effects through inhibition of P53-PUMA signaling. An increase in p53 mRNA and protein was observed in BM Lin^−^c-Kit^+^ cells after 200 cGy irradiation, which was significantly suppressed by IL-33 treatment (Fig. 4c). IL-33 repressed radiation-induced BM Lin^−^c-Kit^+^ cell apoptosis but had no effect on those pretreated with Pifithrin-α (PFTα), a p53 inhibitor (Fig. 4d). PUMA expression enhanced apparently in BM HSCs after 200 cGy irradiation, where PUMA mRNA and protein expression did not change in irradiated PFTα-pretreated HSCs, implying that PUMA induction in HSCs is p53 dependent (Fig. 4e). IL-33 treatment inhibited radiation-induced PUMA expression in BM Lin^−^c-Kit^+^ cells but had no effect on PFTα-pretreated Lin^−^c-Kit^+^ cells (Fig. 4e).

Bone marrow Lin^−^c-Kit^+^ cells with specific PUMAα knockdown showed a lower percentage of apoptotic cells and increased CFC content at 72 h after 200 cGy irradiation compared to PUMA-expressing bone marrow Lin^−^c-Kit^+^ cells (Fig. 4f, g). IL-33 treatment increased HSC survival and CFC regeneration in PUMA-expressing Lin^−^c-Kit^+^ cell cultures after irradiation but had no effect on HSC survival or CFC production in PUMA deficient Lin^−^c-Kit^+^ cell cultures after irradiation (Fig. 4f, g). All together, these results suggest that IL-33-mediated inhibition of radiation-induced HSC apoptosis is dependent on inhibition of PUMA.

### ST2 deficiency impairs HSC regeneration after TBI

**Fig. 5:**
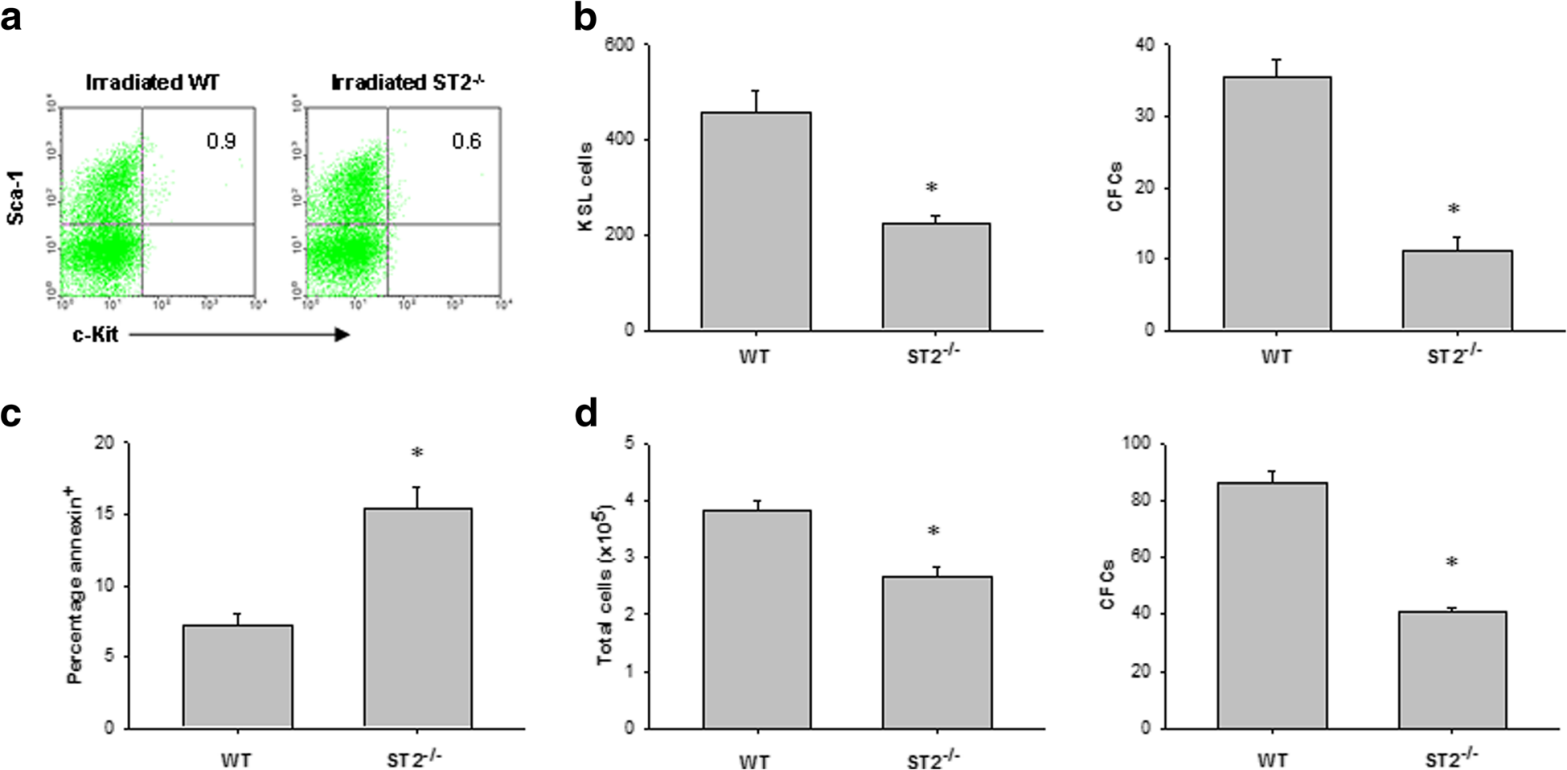
Deficiency of ST2 impairs HSC regeneration after irradiation. **a** Representative FACS analysis of bone marrow c-Kit^+^Sca-1^+^ cells within the Lin^−^ gate (KSL) at day 7 in irradiated WT and ST2-deficient mice. The numbers shown indicate the percentage of c-Kit^+^Sca-1^+^ cells within the Lin^−^population. **b** Bone marrow KSL cells and CFCs at day 7 in irradiated WT and ST2-deficient mice. Data are mean ± SEM (*n* = 6 in each group). *P < 0.05 compared to ST2^−/−^ mice. **c** The percentage of annexin^+^CD45^+^ cells in the bone marrow at day 7 in irradiated WT and ST2-deficient mice. Data are mean ± SEM (*n* = 6 in each group). *P < 0.05 compared to ST2^−/−^ mice. **d** Numbers of total cells and CFCs at 72 h in irradiated bone marrow Lin^−^c-Kit^+^ cells from WT and ST2-deficient mice. Data are mean ± SEM (*n* = 3 in each group). *P < 0.05 compared to ST2^−/−^ TSF

To determine whether ST2 deficiency can suppress HSC regeneration in vivo, we compared the recovery of the bone marrow hematopoietic stem and progenitor cells in WT mice and ST2^−/−^ mice after 550-cGy TBI. At day 7 after TBI, ST2^−/−^ mice significantly decreased the numbers of bone marrow KSL cells and CFCs compared with WT mice (Fig. 5a, b). Meanwhile, ST2^−/−^ mice displayed enhanced numbers of annexin-positive bone marrow hematopoietic cells compared to WT controls (Fig. 5c). We cultured bone marrow Lin^−^c-Kit^+^ cells from WT or ST2^−/−^ mice in cytokine medium for 72 h and found decreased total cell growth and CFC production in the ST2-deficient cells post 200 cGy irradiation (Fig. 5d). Together, these results suggest that ST2 may be necessary for normal bone marrow stem and progenitor cell regeneration after TBI.

## Discussion

There is a variety of studies that IL-33 originally known as a Th2-dominant cytokine performs a beneficial role in the treatment of situations such as transplantation, obesity, and cardiovascular diseases [16, 24, 25]. More recently, several studies suggest that IL-33 also plays an important role in the modulation of hematopoiesis by acting on HSCs in the bone marrow [26]. As is already known, the process of the bone marrow hematopoiesis is complex and HSC regeneration and differentiation is generally implicated in a variety of stromal cells, cytokines, and growth factors [27, 28]. Nevertheless, the underlying mechanisms by which IL-33 is involved in hematopoietic homeostasis remains poorly defined.

This study was conducted to determine the potential effect of IL-33 in a mouse model of radiation-induced hematopoietic damage. Our results have revealed that the level of alarmin IL-33 was quickly elevated in the bone marrow serum after ionizing radiation. IL-33 treatment in turn ameliorated radiation injury to HSCs, and systemic delivery of IL-33 improved the survival of irradiated mice. On the contrary, IL-33 receptor ST2-deficient mice had dramatically decreased the bone marrow HSC and progenitor cell recovery after TBI compared to WT littermates. Notably, the radioprotection of IL-33 in hematopoietic failure was associated with suppression of HSC apoptosis and augmentation of HSC reconstitution.

Ionizing radiation has been well known to primarily cause DNA damage that can lead to a cell-cycle arrest or apoptotic cell death of HSC and progenitor cells in the bone marrow [29]. In the presence of radiation-induced DNA damage, a cell-cycle arrest can occur through p53-dependent or p53-independent mechanisms [30, 31]. DNA damage-induced growth arrest of hematopoietic cells can be overridden by treatment with cytokines such as IL-3 or erythropoietin (EPO) [32, 33], and cytokine-mediated induction of HSC and progenitor cell survival and proliferation early post radiation exposure may contribute to short-term hematopoietic recovery and improved near-term survival [34]. Although the precise mechanism behind these effects remains unclear, cytokine treatment may induce synchronous entry of HSC and progenitor cells into the late S phase, a more radioresistant phase of the cell cycle [35]. In nonhematopoietic tissues, IL-33 receptor ST2 has been reported to mediate cell proliferation through activation of the PI3K-AKT pathway [22]. Here, we observe that IL-33 treatment overrides DNA damage-induced growth arrest by promoting early HSC cycling after radiation exposure and that this effect is dependent on activation of the PI3K-AKT signaling. These results suggest that IL-33-mediated induction of HSC proliferation facilitates the early recovery of the hematopoietic progenitor pool after irradiation.

The p53-upregulated modulator of apoptosis (PUMA) was originally recognized as a transcriptional target of p53 and a mediator of DNA damage-induced apoptosis [36, 37]. PUMA appears to be essential for hematopoietic cell death triggered by ionizing radiation, deregulated *c-Myc* expression, and cytokine withdrawal [23]. Deletion of PUMA protects HSC and progenitor cells from radiation-induced death and confers a striking survival advantage to irradiated animals [38]. We demonstrate in the present study that PUMA is a powerful executor of p53-mediated apoptosis in HSCs after irradiation. IL-33 treatment suppresses radiation-induced upregulation of PUMA in HSC and progenitor cells. Moreover, the effects of IL-33, mediating radioresistance in HSC and progenitor cells, are dependent largely on the repression of PUMA transcription. These data are in line with the previously published results showing that cytokines such as IL-3 can repress PUMA expression in hematopoietic cells and that cytokine withdrawal mediates hematopoietic cell death in a PUMA-dependent manner [23, 39].

## Conclusions

Our presented data reveal a previously unknown function of IL-33 in promoting HSC regeneration after radiation-caused myelosuppression. We show that bone marrow HSCs express functional IL-33 receptor ST2 and that IL-33 acts directly on HSCs to increase HSC cycling and recovery after ionizing irradiation. Translationally, these findings imply that IL-33 may represent a new therapeutic target both in patients undergoing stem cell transplantation and victims of acute radiation sickness.

## Abbreviations

ARS: Acute radiation syndrome
CFCs: Colony-forming cells
CFU-S12: Colony-forming unit-spleen day 12
ELISA: Enzyme-linked immunosorbent assay
EPO: Erythropoietin
HSC: Hematopoietic stem cell
IL-33: Interleukin-33
IR: Ionizing radiation
KSL: c-Kit^+^Sca-1^+^Lin^−^
MOF: Multi-organ failure
MOI: Multiplicities of infection
PBS: Phosphate-buffered saline
PI3K: Phosphatidylinositol- 4,5-bisphosphate 3-kinase
PUMA: p53-Upregulated modulator of apoptosis
RT-qPCR: Real-time quantitative PCR
TBI: Total body irradiation
